# The crystal structure of the TonB-dependent transporter YncD reveals a positively charged substrate-binding site

**DOI:** 10.1107/S2059798320004398

**Published:** 2020-04-27

**Authors:** Rhys Grinter, Trevor Lithgow

**Affiliations:** a Infection and Immunity Program, Biomedicine Discovery Institute and Department of Microbiology, Monash University, Clayton, VIC 3800, Australia; b School of Biological Sciences, Monash University, Clayton, VIC 3800, Australia

**Keywords:** YncD, Gram-negative bacteria, TonB-dependent transporter, membrane transport, X-ray crystallography

## Abstract

The outer membrane of Gram-negative bacteria is highly impermeable to hydrophilic molecules of larger than 600 Da, protecting these bacteria from toxins present in the environment. In order to transport nutrients across this impermeable membrane, Gram-negative bacteria utilize a diverse family of outer-membrane proteins called TonB-dependent transporters. The majority of the members of this family transport iron-containing substrates. However, it is becoming increasingly clear that TonB-dependent transporters target chemically diverse substrates. In this work, the structure and phylogenetic distribution of the TonB-dependent transporter YncD are investigated. It is shown that while YncD is present in some enteropathogens, including *Escherichia coli* and *Salmonella* spp., it is also widespread in Gammaproteobacteria and Betaproteo­bacteria of environmental origin. The structure of YncD was determined, showing that despite a distant evolutionary relationship, it shares structural features with the ferric citrate transporter FecA, including a compact positively charged substrate-binding site. Despite these shared features, it is shown that YncD does not contribute to the growth of *E. coli* in pure culture under iron-limiting conditions or with ferric citrate as an iron source. Previous studies of transcriptional regulation in *E. coli* show that YncD is not induced under iron-limiting conditions and is unresponsive to the ferric uptake regulator (Fur). These observations, combined with the data presented here, suggest that YncD is not responsible for the transport of an iron-containing substrate.

## Abstract 2

In this work, the structure and the phylogenetic distribution of the outer-membrane transporter YncD are determined, showing that YncD is unlikely to transport an iron-containing substrate.

## 1. Introduction   

The outer membrane of Gram-negative bacteria represents a formidable permeability barrier to hydrophilic molecules of larger than ∼600 Da (van den Berg *et al.*, 2015 ▸). Hydrophilic molecules smaller than this diffusion limit cross the outer membrane through proteins called porins, which can be either promiscuous or substrate-specific (Vergalli *et al.*, 2019 ▸). To import molecules that are too large for diffusion through outer-membrane porins, bacteria employ a diverse family of membrane proteins termed TonB-dependent transporters (TBDTs; Noinaj *et al.*, 2010 ▸). These transporters in the outer membrane are so named because they engage in active transport, the energy for which is provided by the TonB–ExbBD complex in the inner membrane, which spans the periplasm to interact with TBDTs (Maki-Yonekura *et al.*, 2018 ▸; Celia *et al.*, 2016 ▸). TBDTs are specific for a particular substrate, which they bind with high affinity in an extracellular binding pocket (Grinter & Lithgow, 2019*a* ▸,*b* ▸). This high-affinity binding allows TBDTs to capture molecules that are of low abundance in the environment, enabling bacteria to scavenge scarce nutrients (Grinter & Lithgow, 2019*a* ▸). One such scarce nutrient is iron, which is the substrate of the majority of characterized TBDTs (Noinaj *et al.*, 2010 ▸). Most iron-transporting TBDTs bind Fe^3+^ ions in complex with siderophores, organic molecules that bind metal ions with high affinity and are secreted by the bacteria or by competitors from the microbial community (Chu *et al.*, 2010 ▸). In addition to importing Fe–siderophore complexes, some TBDTs harvest iron directly from proteins (Grinter *et al.*, 2016 ▸; Noinaj *et al.*, 2012 ▸). These protein-binding TBDTs often target host proteins and are employed by pathogens to obtain iron during infections (Leduc *et al.*, 2008 ▸; Cornelissen *et al.*, 1998 ▸).

While many of the most intensively studied TBDTs import iron-containing substrates, it is becoming increasingly clear that the members of the TBDT protein family are responsible for the import of chemically diverse substrates (Schauer *et al.*, 2008 ▸). Non-iron TBDT substrates range in size from free metal ions, such as Cu^2+^ and Zn^2+^, to cobalamin, polysaccharides, polypeptides and small globular proteins (Grinter *et al.*, 2012 ▸, 2014 ▸, 2016 ▸, 2018 ▸; Chimento *et al.*, 2003 ▸; Yoneyama & Nakae, 1996 ▸; Calmettes *et al.*, 2015 ▸; Bolam & van den Berg, 2018 ▸; Glenwright *et al.*, 2017 ▸; Madej *et al.*, 2019 ▸). Further, a recent study showed that a distant TBDT family member functions in reverse, mediating the export of a protease substrate (Gómez-Santos *et al.*, 2019 ▸). As functionally characterized TBDTs represent a small segment of this protein family, the extent of the substrate diversity of this family remains to be discovered.

The model bacterium *Escherichia coli* K12 possesses nine TBDTs which target different substrates for import (Grinter & Lithgow, 2019*b* ▸). The TBDTs FepA, CirA and Fiu transport Fe^3+^-containing catecholate siderophores (Grinter & Lithgow, 2019*b* ▸; Buchanan *et al.*, 1999 ▸; Nikaido & Rosenberg, 1990 ▸), FhuA and FhuE transport Fe^3+^-containing hydroxamate siderophores of fungal origin (Grinter & Lithgow, 2019*a* ▸; Pawelek *et al.*, 2006 ▸) and FecA transports ferric citrate (Fe-citrate; Ferguson *et al.*, 2002 ▸), while BtuB targets cobalamin (Chimento *et al.*, 2003 ▸). Recent research suggests that the TBDT YddB from *E. coli* K12 functions in the import of a small iron-containing protein, although the exact nature of this substrate remains to be identified (Grinter *et al.*, 2019 ▸). The final TBDT possessed by *E. coli* K12 is YncD, and the substrate of this transporter remains unknown. The homolog of YncD in *Salmonella enterica* ssp. *enterica* serovar Typhi (*Salmonella* Typhi) has been shown to be important for virulence in a mouse model of infection (Xiong *et al.*, 2012 ▸). As such, the characterization of YncD is important for understanding infections caused by *Salmonella* and other entero­bacterial pathogens that possess it.

In this study, we investigated the structure and the phylogenetic distribution of YncD. Using a comparative genomics approach, we show that YncD is present in members of the Enterobacteriaceae that form commensal or pathogenic associations with the human intestine, and is more broadly present in environmental proteobacterial isolates, including species which are opportunistic pathogens of humans. We determined the crystal structure of YncD, demonstrating that it possesses a compact positively charged extracellular substrate-binding site that is characteristic of TBDTs that import negatively charged small-molecule substrates. Finally, using an *E. coli* strain deficient in all iron-transporting TBDTs, we show that YncD does not play a role in iron acquisition under laboratory conditions, providing further evidence that this transporter does not transport an iron-containing substrate.

## 2. Methods   

### 2.1. Identification and analysis of YncD homologues   

To determine the evolutionary relationship between YncD and other TBDTs of known structure and/or function, their sequences were classified by an all-against-all *BLAST* clustering algorithm based on pairwise similarities. The resulting data set was visualized with *CLANS* with an *E*-value cutoff of 1 × 10^−10^ (Frickey & Lupas, 2004 ▸).

To identify YncD sequences in available bacterial genomes, an *HMMER* search was performed on the UniProt reference proteomes database using YncD as the search sequence (Finn *et al.*, 2011 ▸; Chen *et al.*, 2011 ▸). An *E*-value cutoff of 1 × 10^−50^ was applied to hits. This search yielded a total of 568 YncD homologue sequences. These sequences were classified by an all-against-all *BLAST* clustering algorithm based on pairwise similarities. The resulting data set was visualized with *CLANS* with an *E*-value cutoff of 1 × 10^−130^. Sequence clusters were identified in *CLANS* using a network-based algorithm, with a minimum group size of ten (Frickey & Lupas, 2004 ▸). The largest sequence cluster from this clustering consisted of 359 sequences, including YncD from *E. coli*, and was designated the YncD group. This group was isolated and was subjected to further clustering with an *E*-value cutoff of 0. A multiple sequence alignment was performed on 331 full-length sequences from the YncD group using the *Clustal* algorithm (Larkin *et al.*, 2007 ▸). The environment or host of isolation for a subset of YncD sequences (105 sequences) was determined from genome metadata from the Ensembl and UniProt databases (Hubbard *et al.*, 2002 ▸; Apweiler *et al.*, 2004 ▸).

### 2.2. Expression and purification of YncD   

The open reading frame encoding YncD was amplified by PCR from *E. coli* BW25113 using primers containing 5′ BamHI and 3′ XhoI restriction sites (forward, CCATCGGATCCGGCTGATGAACAGACTATGATTGTC; reverse, CCATCCTCGAGTTACTCAAATCTCCACGCAATATTCAT). This YncD open reading frame was then cloned into a modified pET-20b vector with a PelB signal sequence followed by an N-terminal 10×His tag and Tobacco etch virus (TEV) protease cleavage site via restriction digestion and ligation. The resulting vector was transformed into *E. coli* BL21 (DE3) C41 cells (Miroux & Walker, 1996 ▸). Protein expression was performed in Terrific Broth (12 g tryptone, 24 g yeast extract, 61.3 g K_2_HPO_4_, 11.55 g KH_2_PO_4_, 10 g glycerol) with 100 mg ml^−1^ ampicillin for selection. The cells were grown at 37°C until the OD_600_ reached 1.0, induced with 0.3 m*M* isopropyl β-d-1-thiogalactopyranoside (IPTG) and grown for a further 14 h at 25°C. The cells were harvested by centrifugation and lysed using a cell disruptor (Emulsiflex) in lysis buffer (50 m*M* Tris, 200 m*M* NaCl pH 7.9) in the presence of 0.1 mg ml^−1^ lysozyme, 0.05 mg ml^−1^ DNAse I and cOmplete protease-inhibitor cocktail tablets (Roche).

The resulting lysate was clarified by centrifugation at 10 000*g* for 10 min and the supernatant from this low-speed spin was then centrifuged for 1 h at 100 000*g* to isolate a membrane fraction. The supernatant was decanted and the membrane pellet was suspended in lysis buffer using a tight-fitting homogenizer. The resuspended membranes were solubilized by the addition of 10% Elugent (Santa Cruz Biotechnology) and incubated with gentle stirring at room temperature for 20 min. The solubilized membrane-protein fraction was clarified by centrifugation at 20 000*g* for 10 min. The supernatant containing the solubilized proteins was applied to Ni-agarose resin equilibrated in Ni-binding buffer DDM [50 m*M* Tris, 500 m*M* NaCl, 20 m*M* imidazole, 0.03% dodecylmaltoside (DDM) pH 7.9]. The resin was washed with 10–20 column volumes of Ni-binding buffer DDM before elution of the protein with a step gradient of 10%, 25%, 50% and 100% Ni gradient buffer DDM (50 m*M* Tris, 500 m*M* NaCl, 1 *M* imidazole, 0.03% DDM pH 7.9). YncD eluted in the 50% and 100% gradient steps. Eluted fractions containing YncD were pooled and applied onto a Superdex S200 26/600 size-exclusion column equilibrated in SEC buffer DDM (50 m*M* Tris, 200 m*M* NaCl, 0.03% DDM pH 7.9). To exchange YncD into the detergent octyl β-d-glucopyranoside (βOG) for crystallographic and biochemical analysis, SEC fractions containing YncD were pooled and applied to Ni-agarose resin equilibrated in βOG buffer (50 m*M* Tris, 200 m*M* NaCl, 0.8% βOG pH 7.9). The resin was washed with ten column volumes of βOG buffer before elution with βOG buffer plus 250 m*M* imidazole. Fractions containing YncD were pooled and 6×His-tagged TEV protease (final concentration 2 mg ml^−1^) and DTT (final concentration 1 m*M*) were added. This solution was then dialysed against βOG buffer for 4–6 h at 20°C to allow TEV protease cleavage of the 10×His tag from YncD and the removal of excess imidazole. The sample was then applied onto Ni-agarose resin to remove TEV protease and the cleaved histidine-containing peptide. The flowthrough containing YncD from this step was collected, concentrated to 10 mg ml^−1^ in a 30 kDa cutoff centrifugal concentrator, snap-frozen and stored at −80°C.

### 2.3. Crystallization, data collection and structure solution of YncD   

Purified YncD in βOG was screened for crystallization conditions using commercially available screens (approximately 600 conditions). Crystals grew in a number of conditions, with a condition consisting of 0.2 *M* calcium acetate, 0.10 *M* Tris pH 8.5, 20% PEG 3000 chosen for optimization. Concentration and pH grid screening was performed, yielding an optimized crystallization condition consisting of 0.17 *M* calcium acetate, 0.10 *M* Tris pH 7.0, 16% PEG 3000. Crystals from this condition were looped, the crystallization solution was removed by wicking and the crystals were flash-cooled in liquid nitrogen. Diffraction data were collected at 100 K at the Australian Synchrotron and were processed in space group *P*2_1_22_1_. The data were anisotropic, with spot streaking observed (Supplementary Fig. S6); diffraction extended to approximately 2.7, 3.5 and 3.2 Å resolution along the *h*, *k* and *l* axes, respectively. The data were manually processed using *XDS*, scaled using *XSCALE* and anisotropically truncated using the diffraction anisotropy server (Strong *et al.*, 2006 ▸; Kabsch, 2010 ▸). The final data set was truncated to 2.96, 3.5 and 3.4 Å resolution along the *h*, *k* and *l* axes, respectively (Table 1 ▸). A molecular-replacement model was generated by inputting the structure of the closest YncD homologue, FecA (PDB entry 1kmo; Ferguson *et al.*, 2002 ▸), and the amino-acid sequence of YncD into *Sculptor* from the *Phenix* package (Liebschner *et al.*, 2019 ▸). Using this model, a molecular-replacement solution was obtained using *Phaser* from the *Phenix* package, with a TFZ score of 8.1 and an LLG of 161. However, with the quality of the data, the phasing power from this model was insufficient for further model building and refinement. As a result, soaking was performed to obtain a heavy-atom derivative. Using a fine acupuncture needle, a small quantity of potassium tetranitroplatinate salt was added to the drop containing the YncD crystals, the well was resealed and the crystals were incubated for 60 min. These crystals were then looped, the crystallization solution was removed by wicking and the crystals were flash-cooled in liquid nitrogen. Data were collected from the potassium tetranitroplatinate-soaked crystals at 100 K at the Australian Synchrotron using a wavelength of 0.987 Å; the crystals diffracted anisotropically to 3.5, 4.9 and 4.2 Å resolution along the *h*, *k* and *l* axes, respectively. To preserve the anomalous differences, no anisotropic correction was performed on these data, leading to poor statistics in the outer diffraction shell (Table 1 ▸). A heavy-atom search was performed using SAD in *SHELX* within the *CCP*4 software package (Sheldrick, 2008 ▸; Winn *et al.*, 2011 ▸). Three heavy-atom sites were identified, which were provided to *phenix.autosol* within the *Phenix* package along with the molecular-replacement solution for the YncD homology model for phasing and density modification (Adams *et al.*, 2010 ▸). Using the YncD homology model as a starting model, an initial structure of YncD was constructed using these experimentally phased density-modified maps with *Coot* (Emsley *et al.*, 2010 ▸). This model was used for molecular replacement of the 2.96–3.5 Å resolution native data using *Phaser* (McCoy *et al.*, 2007 ▸). The model of YncD was then built using *Coot* and was refined using the *Phenix* package and *BUSTER* (Emsley *et al.*, 2010 ▸; Adams *et al.*, 2010 ▸; Smart *et al.*, 2012 ▸).

### 2.4. *E. coli* Δ*yncD* mutant generation and growth analysis   

The *E. coli* BW25113 Δ*yncD* mutant strain was created using the λ Red system (Datsenko & Wanner, 2000 ▸). A kanamycin-resistance (KanR) cassette flanked by 300 bp of genomic DNA either side of the chromosomal location of *yncD* was amplified by PCR (forward primer, AAACAGGCTATTTCGCTTAGCGA; reverse primer, GAACCTAACAGTAATGAACCACG) using the specific mutant from the *E. coli* Keio collection (Baba *et al.*, 2006 ▸) as a template, generating the *yncD*-*kan* KO cassette. *E. coli* ΔTBDT cells (Grinter & Lithgow, 2019*a* ▸) were transformed with the λ Red recombinase plasmid pKD46 (Datsenko & Wanner, 2000 ▸) and grown at 30°C (LB broth + 100 µg ml^−1^ ampicillin + 100 µ*M* FeSO_4_) to an OD_600 nm_ of 0.1 before λ Red recombinase was induced by the addition of 0.2% l-arabinose. The culture was then grown at 30°C until an OD_600 nm_ of 0.6–0.8 was attained, and the cells were transformed with the *yncD*-*kan* KO cassette using the room-temperature electroporation method (Tu *et al.*, 2016 ▸). Briefly, bacterial cells were isolated by centrifugation at 3000*g* for 3 min and washed twice with a volume of sterile 10% glycerol equal to the volume of culture used. The cells were then resuspended in 10% glycerol to a volume of 1/15 of that of the culture. The *yncD*-*kan* KO cassette DNA (100–500 ng) was then added to 100 µl of the resuspended bacteria and the mixture was electroporated. 1 ml LB broth was added to the cells post-electroporation and the culture was recovered at 37°C for 1 h before plating onto LB agar + 30 µg ml^−1^ kanamycin + 100 µ*M* FeSO_4_. PCR was used to validate that the colonies did indeed have the KanR cassette in place of the gene of interest.

To remove the KanR gene and generate a ‘clean’ *yncD* deletion, the mutant strain was transformed with the plasmid pCP20 (Doublet *et al.*, 2008 ▸) containing the ‘flippase cassette’. The cells were grown at 30°C under either ampicillin (100 µg ml^−1^) or chloramphenicol (30 µg ml^−1^) selection to maintain the plasmid. A single colony of the mutant pCP20-containing strain was used to inoculate 1 ml LB broth + 100 µ*M* FeSO_4_ (no selection). The culture was grown overnight at 43°C to activate expression of the flippase gene. This culture was then subjected to tenfold serial dilution in sterile LB broth and plated onto LB agar + 100 µ*M* FeSO_4_ with no selection. The resulting colonies were patched onto LB agar containing kanamycin or chloramphenicol or with no selection. PCR was used to validate colonies that were unable to grow in the presence of kanamycin or chloramphenicol but grew in the absence of selection and had no remnant of the KanR cassette in the deletion of *yncD*.

*E. coli* ΔTBDT, *E. coli* ΔTBDT/*ΔyncD* and wild-type *E. coli* BW25113 cells were grown in LB broth until the stationary phase. These cultures were used to inoculate 20 ml M9 minimal medium in the presence or absence of 10 m*M* citrate or 100 µ*M* 2,3-dihydrobenzoic acid to an OD_600 nm_ of 0.05. The cultures were grown at 37°C with shaking and the rate of growth was quantified by measuring OD_600 nm_ at hourly intervals.

## 3. Results   

### 3.1. The TonB-dependent transporter YncD is widespread in environmental bacteria   

**Figure 1:**
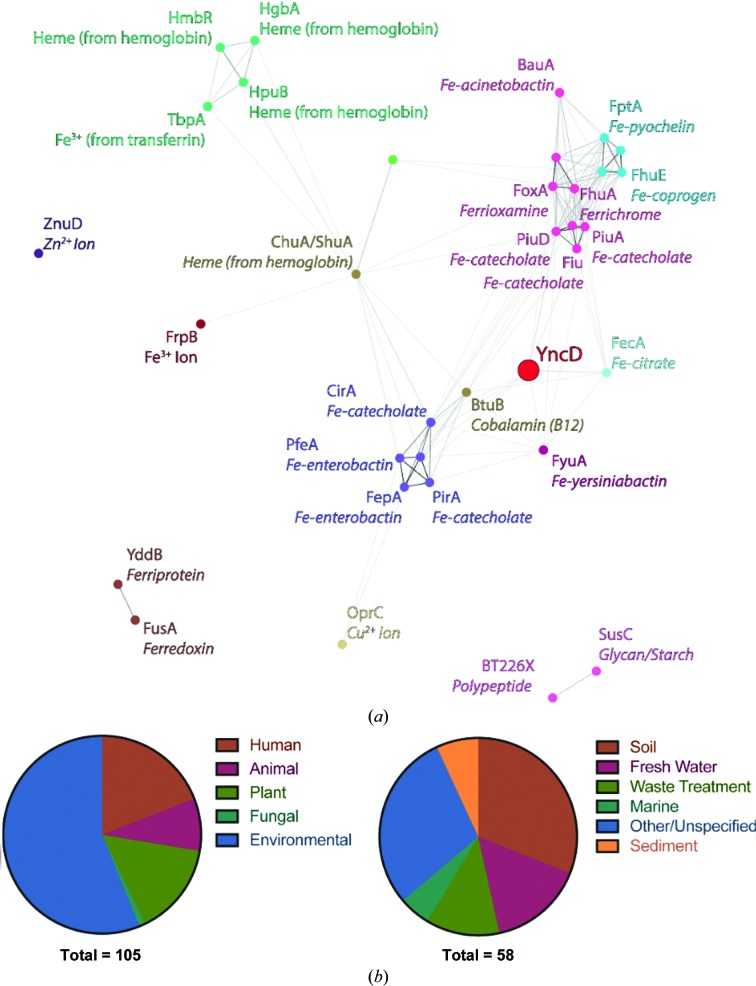
The relationship between YncD and characterized TBDTs and the origin of bacteria that possess YncD. (*a*) Sequence-clustering analysis (*E*-value cutoff = 1 × 10^−10^) of YncD with TBDTs of known structure and/or function. Sequences are color-coded according to the sequence cluster to which they belong. The circle representing the sequence of YncD is enlarged and colored red, showing that YncD does not cluster with the other sequences. (*b*) The environment or host of isolation of a subset of the YncD-containing bacteria summarized in Supplementary Table S1. (*c*) The environment type of YncD-containing bacterial isolates from environmental sources.

To determine the evolutionary relationship between YncD and other functionally characterized TBDTs, we performed a clustering analysis of the amino-acid sequences of these transporters based on pairwise sequence-similarity scores using *CLANS* (Frickey & Lupas, 2004 ▸). This analysis reveals that YncD is distantly related to other TBDTs of known function and does not form a cluster with any of these sequences (Fig. 1 ▸ *a*). This analysis shows that while YncD is most closely related to the TBDTs FecA and BtuB, this reflects only 21% and 18% sequence identity, respectively.

To gain insight into the distribution of YncD in Gram-negative bacteria, we interrogated the UniProt reference proteomes for YncD homologues using the *HMMER* search algorithm (Chen *et al.*, 2011 ▸; Finn *et al.*, 2011 ▸). An initial sequence-similarity *E*-value cutoff of 1 × 10^−50^ was used to capture all YncD sequences present in the database. To remove false-positive non-YncD sequences from this data set, we subsequently clustered the sequences using *CLANS* (Frickey & Lupas, 2004 ▸) with an *E*-value cutoff of 1 × 10^−130^. The largest sequence cluster contained 359 distinct sequences, including YncD from *E. coli*, and was extracted for curation and analysis (Supplementary Table S1 and Fig. S1*a*). Partial sequences were removed from this data set, yielding 331 sequences. These were reclustered with an *E*-value cutoff of 0, revealing several distinct subsets within the YncD protein subfamily (Supplementary Fig. S1*b*). A multiple sequence alignment of these diverse YncD sequences was then performed, revealing that they share amino-acid sequence identities ranging between 33% and 99% (Supplementary Table S2 and Data S1) and highlighting a number of conserved regions. Most notably, the region corresponding to amino acids 79–150 of YncD from *E. coli* is highly conserved among all homologues (Supplementary Fig. S2 and Data S1). This region encompasses the loops of the N-terminal plug domain of the transporter, which are known to mediate substrate binding, suggesting that the YncD homologues identified target a chemically similar substrate.

To explore potential niche-specific functions associated with YncD, we interrogated genome metadata from those bacteria identified in the YncD *HMMER* search. The environment of isolation of 105 YncD-containing species was determined, with all bacteria belonging to either the Gammaproteobacteria or Betaproteobacteria (Supplementary Table S1). While some of these bacterial species were isolated from a human host (largely Enterobacteriaceae associated with the gut or Pseudomonadales associated with respiratory infections), the majority of YncD-containing bacteria were environmental isolates (Fig. 1 ▸ *b*). The environmental isolates originated from soil, sediment or water samples (Fig. 1 ▸ *c*). The widespread distribution of YncD in environmental isolates, and its absence from most specialist bacterial pathogens, is most consistent with YncD playing a role in the acquisition of a molecule produced by the microbial community.

### 3.2. The crystal structure of YncD from *E. coli* reveals a positively charged substrate-binding site   

To gain insight into the evolutionary relationship between YncD and other TBDTs and to provide clues to the nature of the YncD substrate, we solved the crystal structure of YncD by X-ray crystallography. Crystals of YncD from *E. coli* were prepared and diffraction data were collected. The diffraction from YncD crystals exhibited significant anisotropy. Diffraction data were processed and scaled anisotropically using the diffraction anisotropy server with a final maximum resolution of between 2.96 and 3.5 Å, depending on the reciprocal-lattice direction (Table 1 ▸; Strong *et al.*, 2006 ▸). Molecular replacement using a model derived from the closest YncD homologue FecA (PDB entries 1kmo and 1kmp) produced a successful solution (Ferguson *et al.*, 2002 ▸). However, this solution did not provide sufficient phasing power for model improvement. To obtain experimental phases, YncD crystals were soaked with potassium tetranitroplatinate and anomalous diffraction data were collected to 3.5 Å resolution (Table 1 ▸). Phases were obtained for this data set using single-wavelength anomalous dispersion phasing in combination with molecular replacement using the FecA-derived model (MR-SAD). An anomalous substructure of two high-occupancy Pt atoms and one low-occupancy Pt atom was located (Supplementary Fig. S3). An initial model of YncD was built using maps from this data set and was utilized to obtain phases for the higher resolution data set by molecular replacement. The structure of YncD was then built and refined using the anisotropically truncated native data (Table 1 ▸).

**Figure 2:**
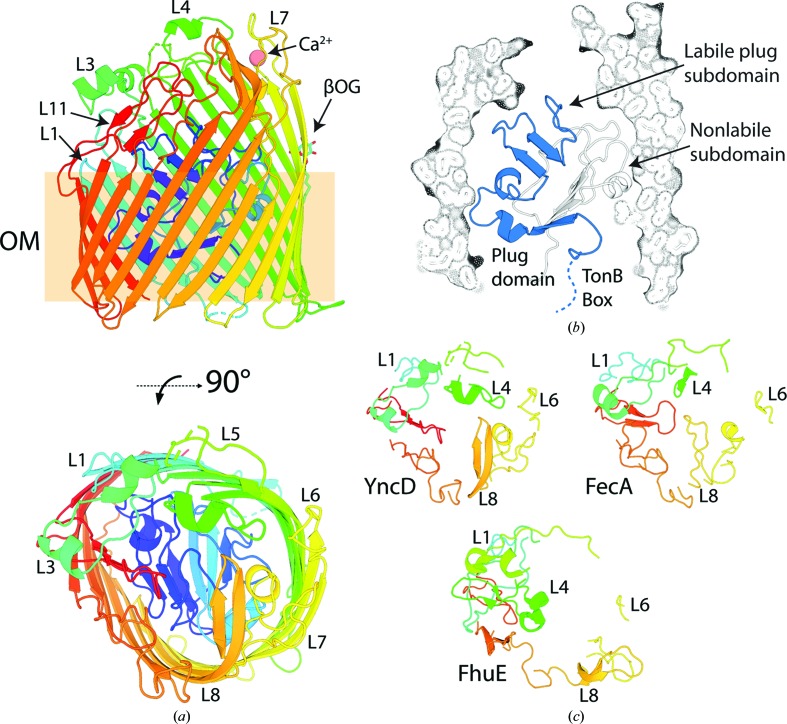
The crystal structure of YncD reveals structural homology to the distantly related transporter FecA. (*a*) The crystal structure of YncD shown as a cartoon representation with rainbow coloring from the N-terminus (blue) to the C-terminus (red). Selected loops are labeled for reference. *i.e.* L3 = loop 3. The outer membrane-embedded region is boxed and labeled ‘OM’. A Ca^2+^ ion and a βOG detergent molecule that co-crystallized with YncD are shown as a sphere and in stick representation, respectively. (*b*) The crystal structure of YncD with the barrel domain shown as a cutaway surface and a stick representation and the plug domain shown as a cartoon representation. The labile section of the plug domain hypothesized to be displaced by TonB is shown in blue (Hickman *et al.*, 2017 ▸), including the TonB box, which is disordered in the structure. (*c*) The extracellular loops of YncD, FecA (PDB entry 1kmp; Ferguson *et al.*, 2002 ▸) and FhuE (PDB entry 6e4v; Grinter & Lithgow, 2019*a* ▸) shown as a cartoon representation, with rainbow coloring and the same orientation as the top-down 90° rotated view in (*a*).

As expected based on other members of the TBDT family, structurally YncD consists of a 22-stranded transmembrane β-barrel which is occluded by a globular N-terminal plug domain (Figs. 2 ▸ *a* and 2 ▸ *b*). The *DALI* server was utilized to search the PDB for structural homologues of YncD (Holm & Laakso, 2016 ▸), revealing that the closest structural homologue to YncD is FecA, which is consistent with our sequence analysis (Supplementary Fig. S3). Despite the low sequence identity of 21% between YncD and FecA, these proteins share a backbone-atom root-mean-square deviation (r.m.s.d.) of 2.2 Å. Consistent with this, the extracellular loop length, secondary and tertiary structure are relatively well conserved between the two proteins compared with the more distantly related TBDT FhuE (Fig. 2 ▸ *c*, Supplementary Fig. S4).

**Figure 3:**
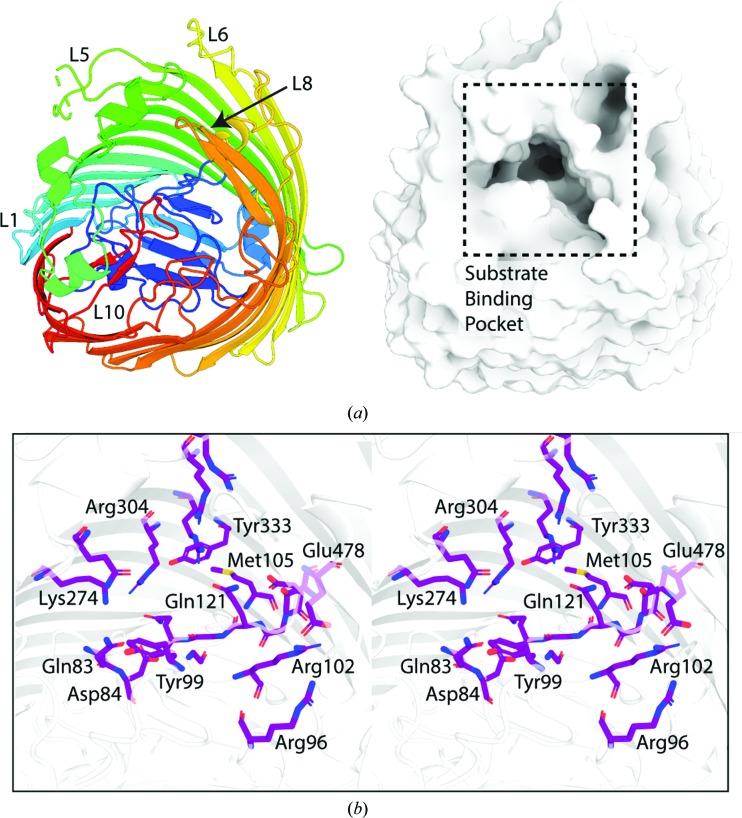

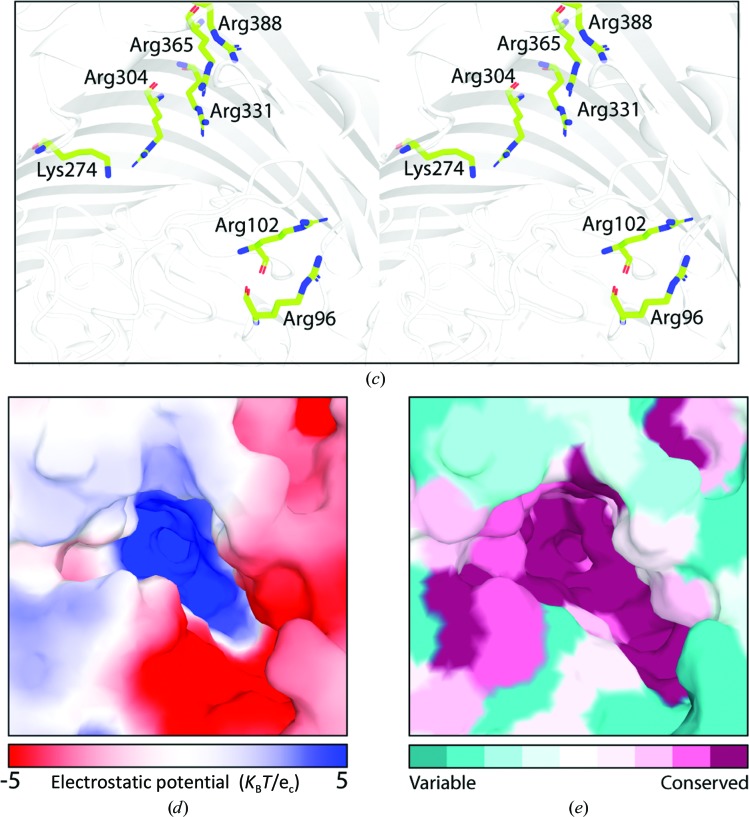
YncD possesses a conserved positively charged substrate-binding pocket. (*a*) A cartoon representation of YncD colored as in Fig. 2 ▸(*a*) (left) and a surface representation in the same orientation showing the substrate-binding pocket (right). (*b*) A stereoview of the amino acids in the YncD substrate-binding pocket which are conserved in YncD homologues according to *ConSurf* (Ashkenazy *et al.*, 2016 ▸). The region shown corresponds to the boxed region in (*a*), YncD is shown as a cartoon representation and conserved residues are shown as sticks. (*c*) A stereoview of positively charged conserved residues in the YncD substrate-binding pocket. (*d*) An electrostatic surface representation of the substrate-binding pocket of YncD. (*e*) A surface representation of the YncD substrate-binding pocket with amino acids color-coded according to their level of conservation across YncD homologues as determined by *ConSurf* (Ashkenazy *et al.*, 2016 ▸). The region of YncD shown in (*d*) and (*e*) corresponds to the boxed region in (*a*).

**Figure 4:**
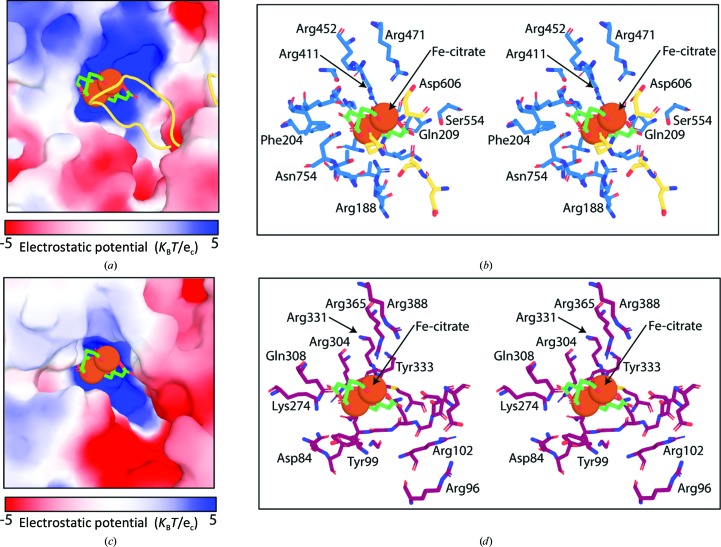
The substrate-binding site of YncD shares similarities in its amino-acid composition and positive charge with that of the ferric citrate transporter FecA. (*a*) An electrostatic surface representation of the substrate-binding pocket of FecA in complex with Fe-citrate. For clarity, the surface of the open conformation of FecA (PDB entry 1kmo) is shown, with the extracellular loops of FecA that undergo a significant conformational change upon Fe-citrate binding shown as a cartoon view (Ferguson *et al.*, 2002 ▸). (*b*) A stereoview of the FecA substrate-binding pocket showing residues involved in the coordination of Fe-citrate that do not change conformation on Fe-citrate binding as blue sticks and residues that change conformation as yellow sticks. (*c*) The electrostatic surface of YncD showing the location of Fe-citrate when the structure of FecA in complex with this ligand is superimposed with YncD. (*d*) A stereoview of the YncD substrate-binding pocket with conserved residues shown as purple sticks and Fe-citrate superimposed as in (*c*). Ferric citrate is shown in a sphere and stick representation in all panels.

A previous analysis of the structures of diverse TBDTs in complex with their substrates revealed the location of a conserved substrate-binding site in these transporters (Grinter & Lithgow, 2019*b* ▸). Based on this analysis, we identified a compact substrate-binding site formed by the extracellular loops of YncD in our structure (Fig. 3 ▸ *a*). Analysis of sequence conservation among YncD homologues using *ConSurf* (Ashkenazy *et al.*, 2016 ▸) and our alignment of YncD sequences revealed that the residues that constitute this binding pocket are well conserved in YncD homologues, suggesting that they play an important role in transporter function (Fig. 3 ▸ *e*, Supplementary Data S1). Many conserved residues in this binding pocket are positively charged or exhibit positive character, including arginine, lysine and glutamine (Figs. 3 ▸ *b* and 3 ▸ *c*). This gives the substrate-binding site of YncD a positive charge, as shown by the calculated electrostatic surface (Fig. 3 ▸ *d*). Electrostatic analysis of the structure of FecA shows that this distant YncD homologue also possesses a positively charged substrate-binding site (Fig. 4 ▸ *a*). In FecA, several arginine and glutamine residues, which give the substrate-binding site its positive character, are responsible for coordinating the carboxylic acid groups of the Fe-citrate complex, and thus are important for substrate binding by FecA (Fig. 4 ▸ *b*; Ferguson *et al.*, 2002 ▸). Superimposition of the FecA–Fe-citrate complex with YncD places the Fe-citrate ligand centrally in the positively charged substrate-binding site of YncD (Figs. 4 ▸ *c* and 4 ▸ *d*). The similarities between the characters of the substrate-binding sites of YncD and FecA suggest that despite their differences they may target chemically similar substrates.

### 3.3. YncD does not import ferric citrate or support the growth of *E. coli* during iron limitation in pure culture   

**Figure 5:**
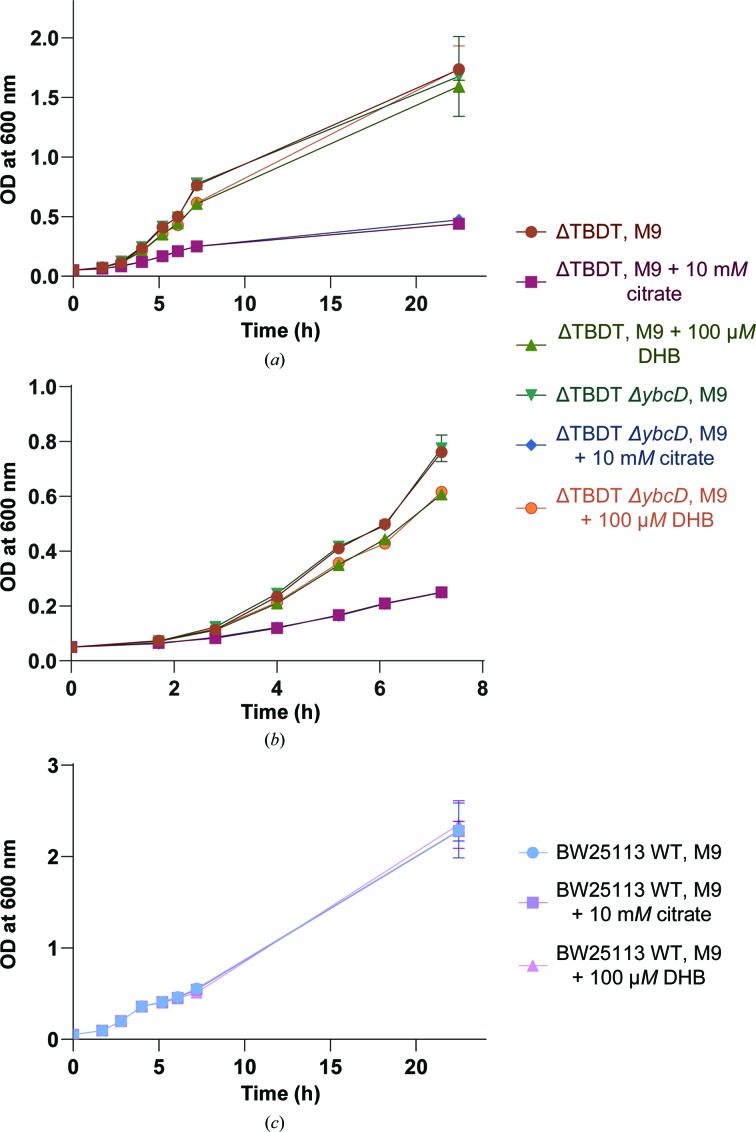
YncD does not import ferric citrate or assist in iron acquisition under laboratory conditions. (*a*) Growth curve of *E. coli* ΔTBDT and *E. coli* ΔTBDT/Δ*yncD* grown in M9 minimal medium in the presence or absence of 10 m*M* citrate or 100 µ*M* 2,3-dihydroxybenzoic acid. (*b*) An enlarged view of the growth curve in (*a*) at hours 1 to 7. (*c*) The growth of wild-type *E. coli* BW25113 under the same conditions as the growth curve shown in (*a*). The error bars represent the standard deviation between three biological replicates.

To test the hypothesis that YncD could support growth under iron-limiting conditions and specifically that it imports ferric citrate, we monitored the growth of an isogenic *E. coli* strain with and without YncD. Because TBDT iron-uptake systems are highly redundant, the *yncD* gene was deleted in a strain that is deficient in the seven TBDTs involved in iron uptake (Supplementary Fig. S5; Grinter & Lithgow, 2019*b* ▸). This ΔTBDT strain grows poorly under iron-limiting conditions (Grinter & Lithgow, 2019*a* ▸), making any additional iron-acquisition defect easier to discern. In M9 minimal medium, the growth of ΔTBDT with or without *yncD* was identical, demonstrating that YncD does not play a role in iron acquisition under these conditions (Fig. 5 ▸ *a*). Since there is some residual free iron in M9 minimal medium, the available iron was chelated by the addition of citrate or the monomeric catecholate compound 2,3-dihydroxybenzoic acid (DHB). As the ΔTBDT strain lacks the outer membrane transporters required to import these substrates, this limits the exogenous iron available to the bacteria. Accordingly, the growth of *E. coli* ΔTBDT was somewhat reduced in the presence of DHB and was drastically reduced in the presence of citrate (Fig. 5 ▸ *b*). Deletion of *yncD* in this strain had no further effect on growth, demonstrating that YncD does not play a role in importing Fe-citrate or Fe-DHB under these conditions (Fig. 5 ▸ *b*). As expected, the growth of wild-type *E. coli* BW25113, which possesses TBDTs for the import of both ferric citrate (FecA) and Fe-DHB (Fiu, CirA and FepA) (Grinter & Lithgow, 2019*b* ▸; Ferguson *et al.*, 2002 ▸), was identical in the presence or absence of these compounds (Fig. 5 ▸ *c*).

## 4. Discussion   

Determination of the function of proteins is one of the most challenging aspects of modern protein biochemistry. The expansion of genomic and metagenomic sequencing data has provided a wealth of protein sequences, most of which are only partially characterized or of completely unknown function. Despite its difficulty, determining the function of novel proteins is invaluable, as without a robust understanding of the functional capacity of the proteome of an organism it is difficult to accurately predict its metabolic capabilities, physiology or lifestyle.

In this work, we show that YncD is a TBDT which is only distantly related to previously characterized members of this family and that YncD is widespread in Gammaproteobacteria and Betaproteobacteria. The crystal structure of YncD shows common structural features with the ferric citrate transporter FecA, but from growth assays YncD appears not to transport ferric citrate. The structure of YncD represents the final structure of the nine TBDTs possessed by the model bacterium *E. coli* K12 (Buchanan *et al.*, 1999 ▸, 2007 ▸; Ferguson *et al.*, 2002 ▸; Locher *et al.*, 1998 ▸; Grinter & Lithgow, 2019*a* ▸,*b* ▸; Grinter *et al.*, 2019 ▸). This structure improves our understanding of the structural variations and similarities that exist in the TBDT family and paves the way for future studies addressing the function of YncD.

Seven of the nine TBDTs present in *E. coli* are involved in iron acquisition. All of these transporters are regulated by the ferric uptake regulator (Fur) and are induced under iron-limiting conditions (Seo *et al.*, 2014 ▸). BtuB, which imports the non-iron-containing substrate cobalamin, is neither regulated by Fur nor induced under iron-limiting conditions (Seo *et al.*, 2014 ▸; McHugh *et al.*, 2003 ▸). Interestingly, like BtuB, YncD is also neither regulated by Fur nor upregulated under iron-limiting conditions, suggesting that it may not function in the import of an iron-containing substrate (McHugh *et al.*, 2003 ▸; Seo *et al.*, 2014 ▸). While the specific substrate transported by YncD remains to be determined, this work provides a number of clues and avenues for further research towards its identification. Based on its compact positively charged extracellular binding site, the substrate of YncD is likely to be relatively small and negatively charged.

As YncD is prevalent in bacterial species from soil and aquatic communities, its substrate is likely to be produced by members of these ecological communities. In addition, the impact of YncD on virulence phenotypes in *Salmonella* Typhi suggest that it is also a molecule that is relevant in a human host context (Xiong *et al.*, 2012 ▸). Based on these considerations and structural parameters in the ligand-binding site, possible substrates for YncD would include molecules containing negatively charged phosphate groups. Other members of the TBDT family have previously been implicated in thiamine pyrophosphate import through comparative genomics (Rodionov *et al.*, 2002 ▸). Alternatively, YncD may import an organic acid-containing compound in complex with a nonferrous metal ion (Ferguson *et al.*, 2002 ▸).

The importance of YncD for virulence in *Salmonella* Typhi (Xiong *et al.*, 2012 ▸) makes understanding the function of this transporter an important question for medical microbiology. Understanding the substrate of YncD and the role of this compound in bacterial virulence will improve our fundamental understanding of infection by this bacterium and will provide a potential target for therapeutic intervention.

## 5. Related literature   

The following reference is cited in the supporting information for this article: Crooks *et al.* (2004 ▸).

## Supplementary Material

PDB reference: outer-membrane transporter YncD, 6v81

Supplementary Data S1. A multiple sequence alignment of YncD sequences from the clustering analysis outlined in Supplementary Tables S1 and S2. For optimal viewing and interpretation, this file should be viewed using a sequence analysis and alignment program, for example ClustalX 2.1. DOI: 10.1107/S2059798320004398/jb5017sup1.txt

Supplementary Table S1. YncD sequences from diverse organisms identified by HMMER search and clustering analysis. DOI: 10.1107/S2059798320004398/jb5017sup2.xlsx

Supplementary Table S2. Sequence-identity matrix of full-length YncD sequences outlined in Supplementary Table S1. DOI: 10.1107/S2059798320004398/jb5017sup3.xlsx

Supplementary Figures and Supplementary Table S3. DOI: 10.1107/S2059798320004398/jb5017sup4.pdf

## Figures and Tables

**Table 1:** Crystallographic data-collection and refinement statistics for YncD Values in parentheses are for the highest resolution shell.

	K_2_Pt(NO_2_)_4_ soak	Native†
Data collection		
Space group	*P*2_1_22_1_	*P*2_1_22_1_
*a*, *b*, *c* (Å)	90.61, 96.28, 127.39	87.94, 98.64, 124.86
α, β, γ (°)	90, 90, 90	90, 90, 90
Wavelength (Å)	0.9763	0.9763
Resolution (Å)	48.14–3.50 (3.83–3.50)	49.32–2.96 (3.14–2.96)
*R*_merge_ (%)	38.6 (665.70)	16.0 (73.7)
*R*_p.i.m._ (%)	11.7 (201.2)	7.2 (36.4)
〈*I*/σ(*I*)〉	7.6 (0.8)	8.4 (2.4)
CC_1/2_	0.999 (0.338)	0.999 (0.727)
Completeness (%)	99.8 (99.5)	75.5 (12.1)
Multiplicity	22.1 (22.5)	6.7 (5.8)
No. of unique reflections	14619 (3395)	17683 (460)
Resolution truncation (reflections with *F*/σ < 3.0 discarded)		
*h*, *k*, *l* (Å)		2.96, 3.5, 3.4
Completeness after truncation (%)		
45.71–3.83 Å		99.9
3.83–2.96 Å		46.3
Refinement statistics		
*R*_work_/*R*_free_ (%)		27.7/31.4
No. of atoms		5106
Average *B* factors (Å^2^)		
Protein		84.09
Ligand		89.40
R.m.s. deviations		
Bond lengths (Å)		0.005
Bond angles (°)		0.787
Ramachandran plot		
Favored (%)		94.25
Allowed (%)		5.28
Outliers (%)		0.47
PDB code		6v81

† Correction applied using the diffraction anisotropy server.
