# Supplementary material for: The crystal structure of the TonB-dependent transporter YncD reveals a positively charged substrate-binding site
Source: Acta Crystallogr D Struct Biol. 2020 Apr 27;76(Pt 5):484–95. doi: 10.1107/S2059798320004398 (PMC7193533; doi:10.1107/S2059798320004398)
Supplement: Supplementary file 4 [file d-76-00484-sup4.pdf]

Supplemental Data

Data S1: A multiple sequence alignment of YncD sequences from clustering analysis outlined in Table S1 and 2. For optimal viewing and interpretation, this file should be viewed using a sequence analysis and alignment program, for example, Clustal X 2.1 [1].

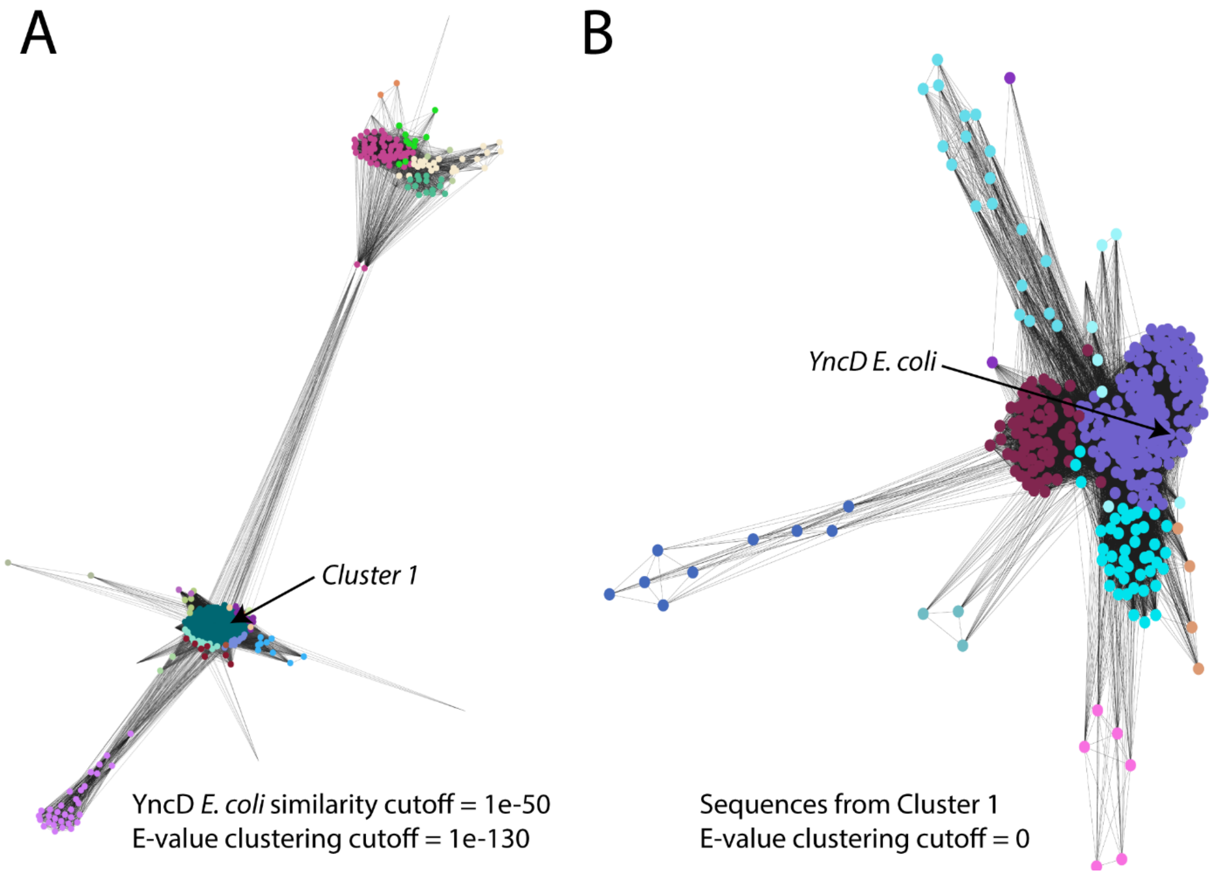

Figure S1 CLANS sequence clustering of YncD homologues from diverse bacterial isolates. (A) CLANS clustering of YncD homologues from the Uniprot references proteomes database identified with an E-value cutoff of $1x10^{-50}$ and clustered based on an E-value cutoff of $1x10^{-130}$ or smaller. (B) YncD sequences from cluster 1 in panel A clustered based on an E-value cutoff of 0.

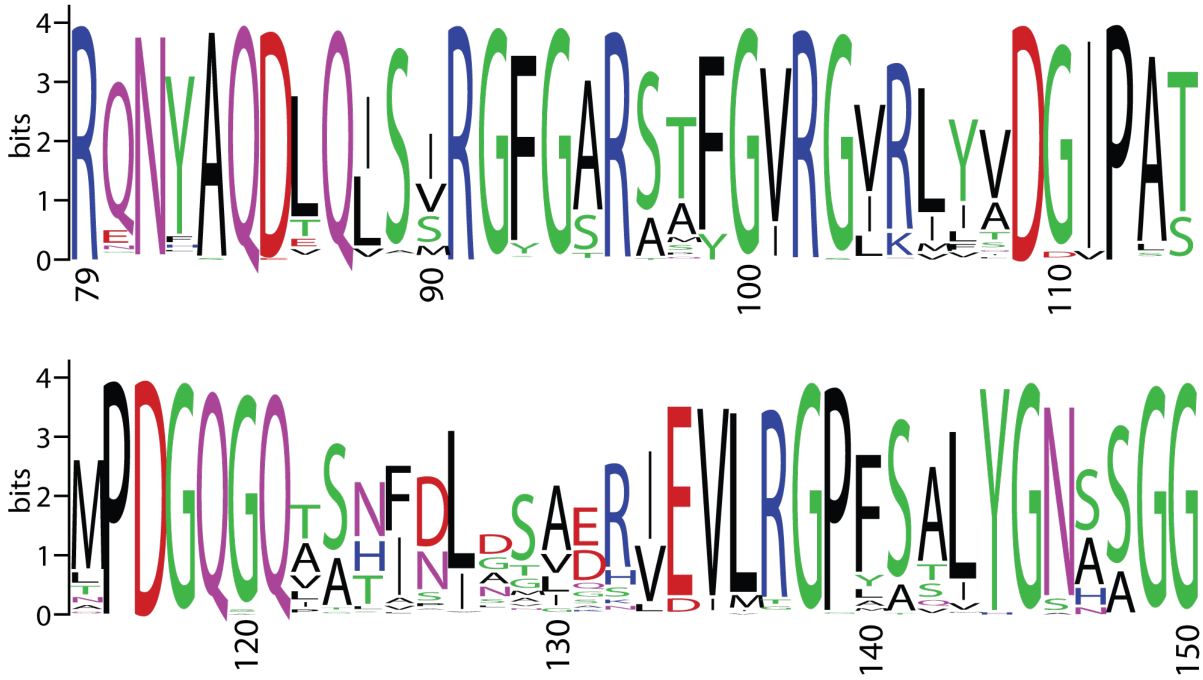

Figure S2 A WebLogo [2] plot showing sequence conservation in YncD family members in the region containing the plug-domain loops that forming the substrate-binding pocket. The number labels on the X-axis correspond to the amino acid sequence number in YncD from *E. coli* BW25113.

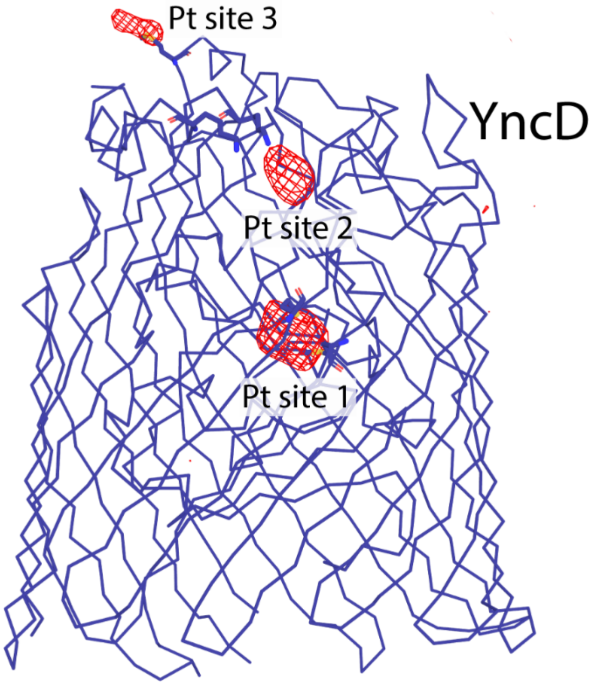

Figure S3 A stereoview of the anomalous difference map derived from diffraction data collected from a potassium tetranitroplatinate soaked YncD crystal. Peaks in the anomalous difference map showing binding sites of tetranitroplatinate molecules are shown as red mesh contoured to 4 σ. Pt sites 1 and 2 represent high occupancy sites > 0.5, and Pt site 3 represents a low occupancy site < 0.2. YncD is shown as a blue ribbon representation.

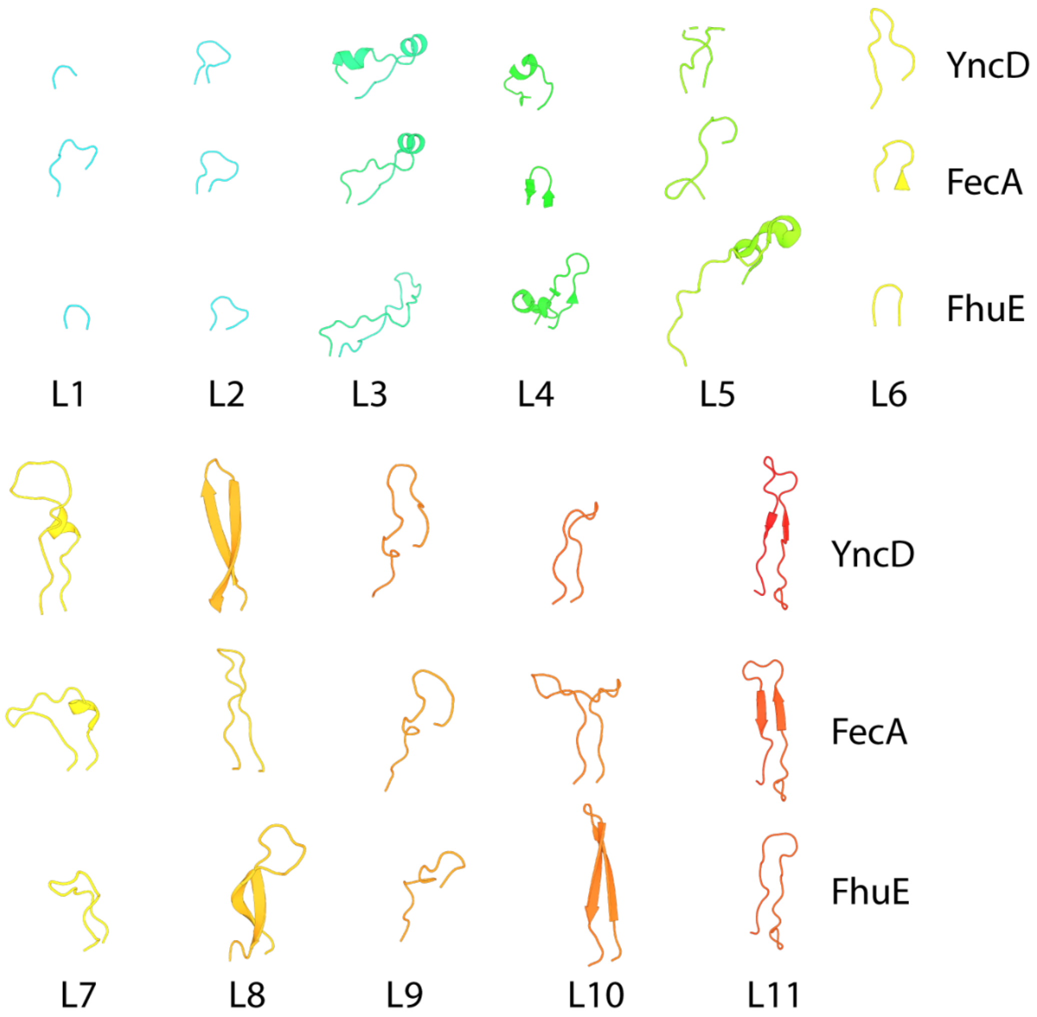

Figure S4 Comparison of the extracellular loops of YncD with other TBDTs. A dissection of the extracellular loops of YncD and the TBDTs FecA and FhuE, providing an indication of the similarity of secondary structure between the extracellular loops of these transporters.

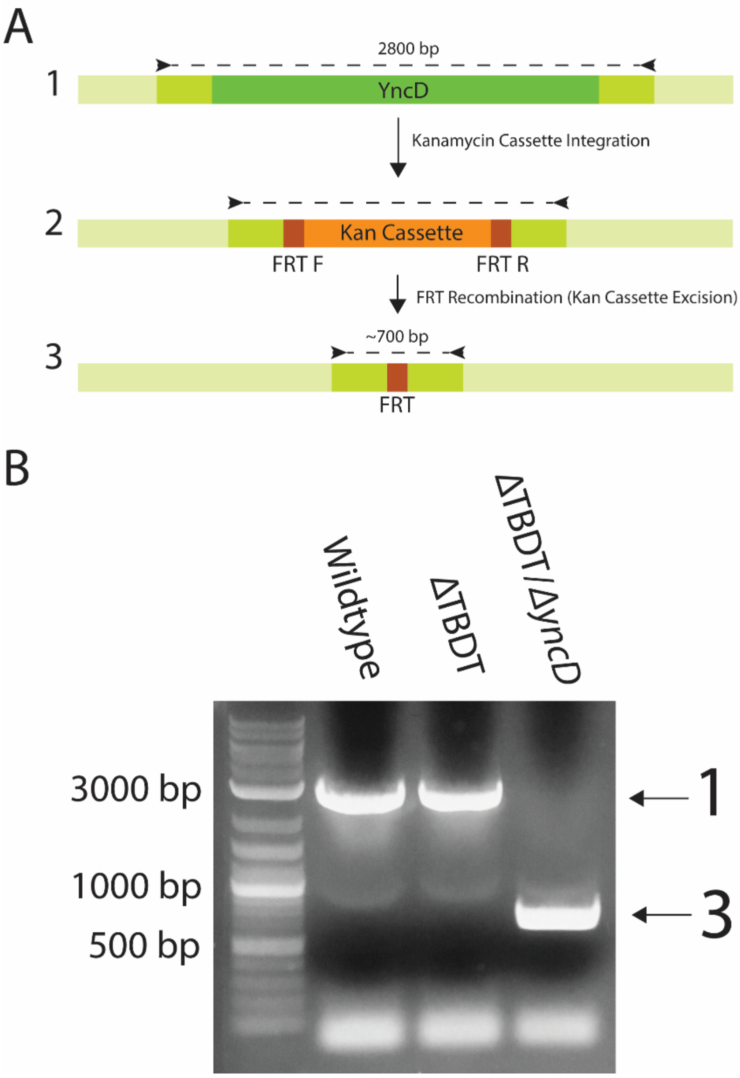

Figure S5 Creation of the *E. coli* ΔTBDT/*ΔyncD* strain. (A) A schematic of the *yncD* knockout process, showing step 1 the original *yncD* gene, step 2 the replacement of the *yncD* gene with a kanamycin resistance cassette (Kan cassette) and step 3 removal of Kan cassette via FRT-site mediated recombination. The primers utilized for Kan cassette amplification and for confirmation of knock out creation are shown as black arrows. (B) PCR amplification from genomic DNA of wildtype, ΔTBDT, and ΔTBDT/*ΔyncD E. coli* strains using *yncD* KO primers shown in panel A, with amplification product corresponding to sizes predicted in the KO scheme. The genome of the ΔTBDT/*ΔyncD* strain was sequenced confirming no non-desired mutations were introduced.

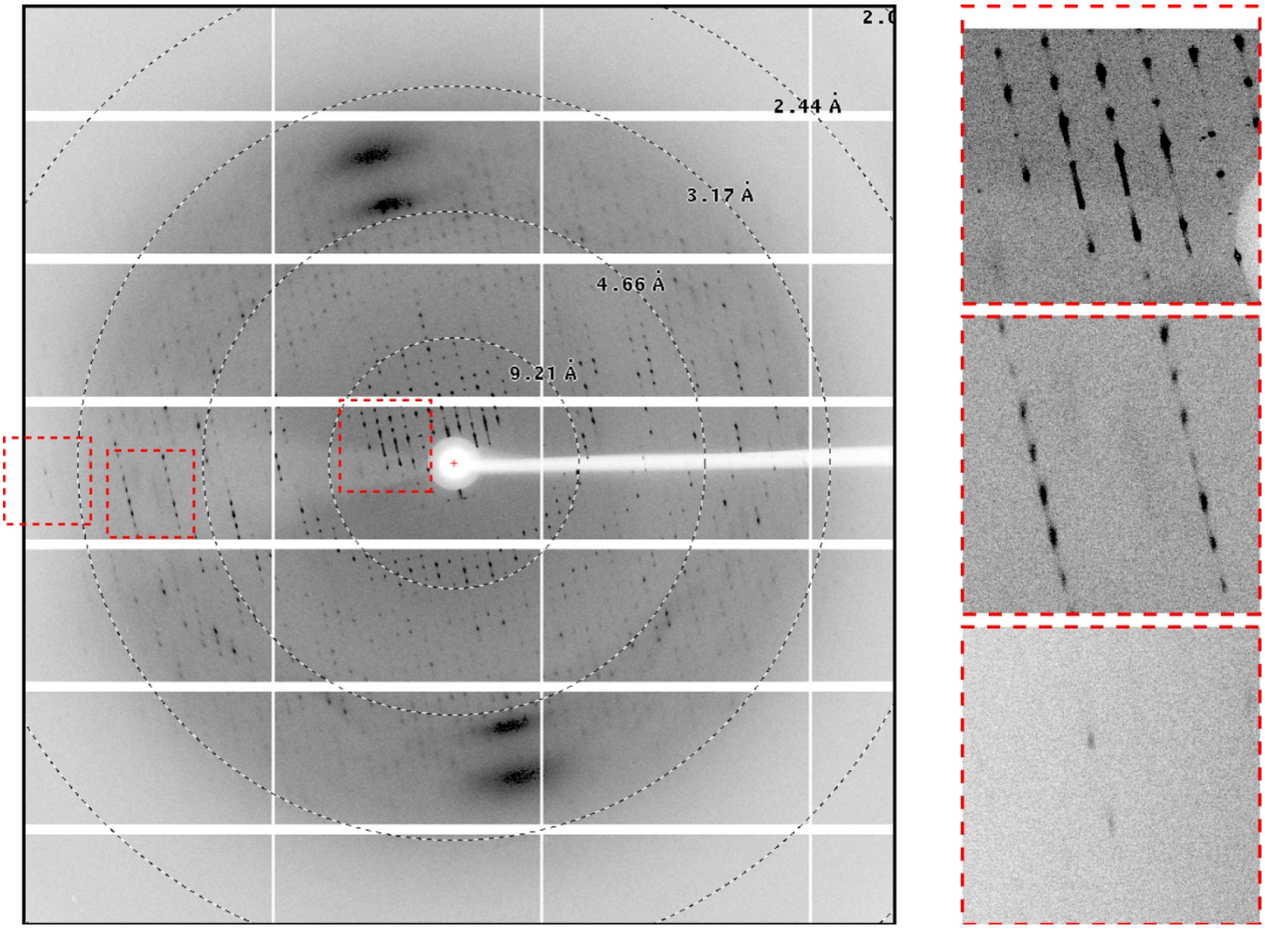

Figure S6 A typical diffraction image from the native YncD dataset. Diffraction from YncD crystals was anisotropic with observable diffraction spots varying from a resolution of ~2.7 to 3.5 Å depending on lattice direction. In addition, diffraction spots displayed a moderate level of streakiness, to which we attribute the relatively high R-values for the refined structure.

Table S1 YncD sequences from diverse organisms identified by HMMER search and clustering analysis (Attached File)

Table S2 Sequence identity matrix of full-length YncD sequences outlined in Table S1 (Attached File)

Table S3 YncD structural homologues identified by the Dali server [3]

Chain	Z	rmsd	lali	nres	%id	Description
1kmo-A	36.6	2.2	596	661	21	Iron(iii) dicitrate transport protein FecA
2gsk-A	36.3	2.7	531	590	18	Vitamin B12 transporter BtuB
4epa-A	34.3	2.7	589	632	20	Ferric-yersiniabactin transporter FyuA
2o5p-A	32.3	2.8	577	772	18	Ferripyoverdine Receptor
1fi1-A	32.2	2.4	589	707	20	Ferrichrome-Iron Receptor
5m9b-A	30.9	3	543	707	19	Ferric-Enterobactin Receptor
5fq6-M	30.8	3.6	573	948	16	SusC
6h7f-A	30.7	2.8	582	674	15	BauA
6fok-A	29.7	3.1	571	651	18	Copper Transporter OprC
6bpm-A	29.6	2.8	581	711	20	Catecholate Siderophore Receptor Fiu
3csl-A	29.2	2.8	568	753	18	HasR
4aip-B	28.4	3.1	557	665	17	FrpB
3v89-A	27.9	3.4	567	853	16	TbpA
5fr8-A	27.8	2.8	542	707	20	Catecholate Siderophore Receptor PirA
4rdr-A	27.7	3.1	556	706	15	ZnuD
4zgv-A	27.2	3.7	558	809	14	Ferredoxin Transporter FusA
6ofr-A	25.5	3.5	556	758	16	Putaative Protein Transporter YddB

References

1. Larkin MA, Blackshields G, Brown N, Chenna R, McGettigan PA, McWilliam H, et al. Clustal W and Clustal X version 2.0. bioinformatics. 2007;23(21):2947-8.
2. Crooks GE, Hon G, Chandonia J-M, Brenner SE. WebLogo: a sequence logo generator. Genome research. 2004;14(6):1188-90.
3. Holm L, Laakso LM. Dali server update. Nucleic acids research. 2016;44(W1):W351-W5.
